# Challenges of maintaining research protocol fidelity in a clinical care setting: A qualitative study of the experiences and views of patients and staff participating in a randomized controlled trial

**DOI:** 10.1186/1745-6215-12-108

**Published:** 2011-05-04

**Authors:** Julia Lawton, Nicholas Jenkins, Julie L Darbyshire, Rury R Holman, Andrew J Farmer, Nina Hallowell

**Affiliations:** 1 Centre for Population Health Sciences, University of Edinburgh, Medical School, Teviot Place, Edinburgh, EH8 9AG, UK; 2 Diabetes Trials Unit, Oxford Centre for Diabetes, Endocrinology, and Metabolism, University of Oxford, OX3 7LJ, UK; 3 National Institute for Health Research, School for Primary Care Research, Department of Primary Health Care, University of Oxford, OX3 7LF, UK; 4 Institute of Health and Society, Newcastle University, Baddiley-Clark Building, Richardson Road, Newcastle upon Tyne, NE2 4AX, UK

## Abstract

**Background:**

Trial research has predominantly focused on patient and staff understandings of trial concepts and/or motivations for taking part, rather than why treatment recommendations may or may not be followed during trial delivery. This study sought to understand why there was limited attainment of the glycaemic target (HbA_1c _≤6.5%) among patients who participated in the Treating to Target in Type 2 Diabetes Trial (4-T). The objective was to inform interpretation of trial outcomes and provide recommendations for future trial delivery.

**Methods:**

In-depth interviews were conducted with 45 patients and 21 health professionals recruited from 11 of 58 trial centres in the UK. Patients were broadly representative of those in the main trial in terms of treatment allocation, demographics and glycaemic control. Both physicians and research nurses were interviewed.

**Results:**

Most patients were committed to taking insulin as recommended by 4-T staff. To avoid hypoglycaemia, patients occasionally altered or skipped insulin doses, normally in consultation with staff. Patients were usually unaware of the trial's glycaemic target. Positive staff feedback could lead patients to believe they had been 'successful' trial participants even when their HbA_1c _exceeded 6.5%. While some staff felt that the 4-T automated insulin dose adjustment algorithm had increased their confidence to prescribe larger insulin doses than in routine clinical practice, all described situations where they had not followed its recommendations. Staff regarded the application of a 'one size fits all' glycaemic target during the trial as contradicting routine clinical practice where they would tailor treatments to individuals. Staff also expressed concerns that 'tight' glycaemic control might impose an unacceptably high risk of hypoglycaemia, thus compromising trust and safety, especially amongst older patients. To address these concerns, staff tended to adapt the trial protocol to align it with their clinical practices and experiences.

**Conclusions:**

To understand trial findings, foster attainment of endpoints, and promote protocol fidelity, it may be necessary to look beyond individual patient characteristics and experiences. Specifically, the context of trial delivery, the impact of staff involvement, and the difficulties staff may encounter in balancing competing 'clinical' and 'research' roles and responsibilities may need to be considered and addressed.

## Background

Type 2 diabetes mellitus is a progressive disorder, with many patients eventually requiring insulin therapy. Alongside management of risk factors such as blood-pressure, smoking and lipids, tight glycaemic control significantly reduces the risks of diabetes-related micro- and macro-vascular complications [1-3]. As a result of these findings, health professionals have, until recently, been encouraged to adopt aggressive approaches to controlling blood glucose levels amongst their type 2 diabetes patients, including early initiation onto insulin.

The 4-T study was a large, 3-year multi-centre, open label, UK-based randomized trial. It was developed because of a lack of clinical knowledge at the time about the most appropriate ways to initiate insulin therapies in patients with type 2 diabetes, and how to help them achieve optimal blood glucose control on intensive insulin treatments. To do this, the trial evaluated and compared the impact of three different licensed analogue insulin regimens in patients amongst whom oral medication was no longer sufficient to maintain good glycaemic control [4,5]. The three insulin regimens investigated were basal (insulin determir, normally taken once a day), biphasic (insulin aspart 30, taken twice daily) and prandial (insulin aspart, taken three times a day), and were all already widely used in routine clinical practice.

The trial employed a treat-to-target approach with a glycaemic target of HbA_1c _≤6.5%, in line with clinical recommendations at the time of trial development [6,7]. This treat-to-target approach was used to help and enable patients to achieve tight blood glucose control and allow the different insulin regimens to be compared in terms of ease of use, acceptability, clinical outcomes (e.g. weight effects, risk of hypoglycemia and impact on microalbuminuria), and patient adherence. A second insulin formulation was added in the second and third years if HbA_1c _levels of ≤6.5% were not achieved. To promote attainment of the trial's glycaemic target, an on online Trial Management System (TMS) was used in all centres. Staff were required to enter patients' clinical data (e.g. weight, self-reported blood glucose readings over a three day period, and reported episodes of hypoglycemia) into the TMS. The TMS then employed a standardized algorithm [8] to determine the insulin doses that needed to be prescribed in order for patients to attain or maintain the glycaemic target, although, in the event of hypoglycaemia or other untoward factors, trial staff were allowed occasional discretion in the prescription of insulin doses. During the trial, patients received a comprehensive monitoring and support package comprising 17 clinic visits and at least 11 telephone contacts over three years. Patients were also encouraged to alter insulin doses themselves in between contacts and in response to self monitoring of blood glucose (SMBG) readings to help attain the trial target. The trial commenced November 2004 and final closeout took place in July 2009.

Despite the intensive input provided during 4-T, there was limited attainment of the glycaemic target across all three treatment groups. At one year, 17% of the biphasic, 24% of the prandial and 8% of the basal group attained an HbA_1c _≤6.5%. At three years, the corresponding proportions were 32%, 45% and 43% [4,5]. Similar outcomes have been observed in other trials involving intensive insulin therapies [9-12] and in routine clinical situations, recent data indicating that control may be sub-optimal in up to 75% of insulin-treated patients [13,14]. This has prompted calls for the behavioral factors to be considered and explored [5]. However, research has predominantly focused on attitudes towards, and barriers to, initiating insulin therapy [15-18]. There is a very limited understanding of how patients and health professionals perceive and respond to tight glycaemic targets and the issues and challenges encountered attaining or sustaining tight glycaemic control after insulin treatment has been initiated. Similarly, while there is an extensive trial literature, this has predominantly focused on patients' motivations for trial participation [19-22] or patient and/or staff understandings of randomization and/or equipoise [23-25], and the implications of these for facilitating recruitment and informed consent [19,26]. Little is known about patients' and health professionals' experiences of trial delivery and why treatments may or may not be adhered to in the context of a clinical trial.

In this paper we report findings of an interview study involving patients and health professionals who had participated in 4-T. This study was conducted to understand why there was limited attainment of the trial target for glycaemic control across all three treatment groups and over time. Key objectives were to inform interpretation of trial outcomes and provide recommendations for future trial delivery.

## Methods

Qualitative methods are recommended when little is known about the area of investigation. In-depth interviews were used in this study as these encourage participants to display their own understandings and meanings, and permit themes and hypotheses which might not have been anticipated to be identified and explored [27].

### Recruitment and sample

Patients and staff were recruited from 11 of the 58 4-T centres across the UK. Centres were selected to ensure diversity in centre size, geographical location and previous trial involvement. Recruitment took place on trial closeout. Patients and staff were sent information packs and invited to opt-in to the study. Sixty-eight patients opted-in of which 45 were interviewed. Patients were selected for interview on the basis of their treatment type, age, gender and glycaemic control, so that the final sample reflected the wider trial population in terms of these characteristics (see Table 1). The patient sample size (n = 45) was determined to allow a diversity of experiences and views to be captured and explored in-depth and for data saturation to occur in key areas (i.e. for no new findings or themes to arise from an analysis of new interviews) [28].

**Table 1:** Patient and staff characteristics

Patients	4T (n = 708)	Qualitative sample (n = 45)
**Age**		
Mean age (± SD)	61.7 (±9.8)*	64.7 (± 8.5)**
		
**Sex**		
Male (%)	456 (64)	29 (64)
Female (%)	252 (36)	16 (36)
		
**Randomisation**		
Biphasic (%)	235 (33)	15 (33)
Prandial (%)	239 (34)	15 (33)
Basal (%)	234 (33)	15 (33)
		
**Glycaemic control at Yr 3**		
Median HbA_1c_	6.9%	6.9%
Number (%) of patients with HbA_1c _£ 6.5%	283 (40)	19 (42)
Number (%) of patients with HbA_1c _£ 7.0%	425 (60)	26 (58)
		

**Health Care Professionals**	**4T ➢**	**Qualitative sample (n = 21)**

**Role**		
Physician (Phy)	-	9
Research Nurse (RNs)	-	12
		
**Experience in diabetic medicine**		
0-5 years	-	1
10+ years	-	8
		
**Clinical background in diabetic nursing prior to 4-T (e.g. Diabetes Specialist Nurse)**		
Yes	-	8
No	-	4
		
**Nurse role during 4-T**		
Research only	-	6
Research and clinical care	-	6

Notes

Percentages rounded up to nearest whole number

* Age at initiation of 4T

** Age at interview

**➢ **Trial data not available

Twenty-one staff members were interviewed, the staff sample including both physicians and research nurses involved in trial delivery and at least one staff member from each centre (see Table 1).

### Data collection & analysis

Interviews took place between October 2008 and July 2009. These were informed by topic guides [see Appendix 1] and conducted at a time and location selected by participants. Interviews lasted 40-120 minutes, were digitally recorded and transcribed verbatim. The study was informed by the principles of grounded theory which involves concurrent data collection and analysis, together with systematic efforts to check and refine developing categories of data [28,29]. Themes and hypotheses identified in early interviews informed questions in later interviews. Team members independently reviewed interview data and regular meetings were held to explore participants' underlying reasoning, discuss deviant cases and reach agreement on recurrent themes and findings. Interviews were coded to capture data relating to the questions and areas explored in the topic guides and emerging findings. QSR Nvivo 2, a qualitative data-indexing package, was used to facilitate data coding and retrieval.

At the point when data collection was stopped no new themes were arising from an analysis of patient and staff interviews. Data presented below are tagged with participants' identifying number and status (P = Patient, RN = Research Nurse, Phy = Physician).

The research was approved by the Hertfordshire Research Ethics Committee (ref: 08/H0311/98). Written informed consent was obtained from patients and staff prior to their interviews. As part of the consent process, patients and staff were informed that study findings would be published in academic journals.

## Results

Findings from the main 4-T trial showed that endpoint glycated haemoglobin were similar for patients randomised to biphasic (median: 7.1%), prandial (median: 6.8%) and basal (median: 6.9%) insulin-based therapies (P = 0.28) [5]. Consistent with these findings, analysis of the qualitative interviews did not reveal widespread or systematic differences in reported adherence amongst patients in the three trial arms. Staff accounts, similarly, did not reveal obvious differences in the treatment, advice and care given to patients in different arms of the trial (hence data reported below cut across the three trial arms). However, whereas patients tended to present relatively unproblematic accounts of participating in, and taking their insulin during, the trial, staff highlighted various difficulties and dilemmas which arose during trial delivery. In particular, staff described experiencing a conflict between their roles and responsibilities as researchers and clinicians. This conflict became most apparent when they were required to treat to the trial's tight glycaemic target.

Below, we begin with patients' experiences of taking part in 4-T before considering staff perspectives and views. We will show that staff accounts need to be considered alongside those of patients to fully understand why there was limited attainment of the trial's glycaemic target.

### Patients' experiences and views

#### Taking insulin during 4-T

As detailed elsewhere [30], virtually all patients described being motivated and committed to taking their insulin during the trial, as they perceived insulin as essential for their diabetes control and long-term health: "if you don't take your medication, then that's it" (P31); "it's a matter of life and death" (P16). Hence, only a minority reported extensive and intentional insulin non-adherence, the most extreme case being P29 who stopped taking insulin for three months after experiencing a severe hypoglycaemic episode requiring emergency services to be called out:

"I'd never had a hypo before, but I'd been feeling funny and I've maybe had a biscuit or something, I've had something to eat and it went away but this Sunday, for some reason, just after lunch, I just went berserk, I nearly wrecked everything in the house. And, of course, the wife panicked and she phoned for the doctor and this emergency doctor came out, because I was ranting and raving on and the wife was panicking." (P29)

While virtually all patients described sometimes forgetting to inject insulin, particularly when daily routines were disrupted, nearly half also talked about occasionally adjusting and/or deliberately skipping doses. In almost all cases, doses were altered or missed as a strategy to curb or avoid hypoglycaemia; for instance, when a meal was skipped or, in P10's case, before undertaking planned physical activity:

"If I'm going walking up the hills, I don't take any, I don't take any Rapid insulin. Either that or I'd be keeling over on the hills because my sugar levels, with doing the extra exercise, my sugar levels would be far too low... So I basically don't take any. And at the end of the day when I come back home, I check my sugar level and it's probably 6.5 or something like that and that's being out on the hills all day, eating as and when I needed." (P10)

Some also described titrating insulin doses according to foods consumed or in response to SMBG readings, to avoid hypoglycaemia or optimise control:

"I now adjust my insulin with the blessing of, eh, my blood sugar level. If they're below six, I use eight units in the morning. If they're above six I use ten. The only one I usually adjust is the morning one, because if I don't, I hypo." (P21)

In almost all cases, patients described discussing these strategies with 4-T staff and receiving their encouragement and support.

#### Titration and intensification of treatment

Patients tended to defer to 4-T staff when decisions were made about titrating and intensifying their insulin. Some also recalled instances when they had been aware of staff not following the TMS' recommendations. Patients described preferring to trust staff's judgement rather than the TMS, because of their holistic knowledge of the patient, their ability to "use common sense" (P11), and patients' experiences of staff taking their personal circumstances into careful consideration:

"Computers are great for maths or something like that but, for making decisions about people's lives, I'd rather have a human view point, that can weigh it up, look at all angles." (P20)

"I'd trust the nurse every time, cos everything was fed into the computer and sent down to Oxford, where as she was seeing me all of the time, what I'm looking like healthwise etc." (P22)

A minority, however, did describe becoming more involved in negotiating their insulin doses with staff as the trial progressed, prompted by experiences of hypoglycaemia which had led them to question or resist TMS recommended doses:

"The information would be put in and generally it would come back and say to me that I needed to increase the dose by quite a substantial amount. But actually the nurse knew I didn't want to do that, that I was wary to do it, and that also I am actually, I believe, very sensitive to insulin, to having hypos... And they were happy to reach a compromise; to discuss how I felt and then agree a path forwards." (P11)

#### Trial targets

Most patients were unaware of the 4-T target for glycaemic control and determined their own success or failure according to staff's encouragement and responses to their HbA_1c _results. In practice, this meant a patient could think they had 'done well' even though they had not reached the trial target, such as P2 who described how, "once it was seven point something, they were all delighted about it." The reverse could also happen, such as when P23 described how she had "felt a failure" despite getting her HbA_1c _down to 6.4%, "because they were always trying for 5.4, 5.6."

### Staff experiences and views

#### Treating to target

All staff expressed ambivalence about the trial target (HbA_1c _≤6.5) because it was tighter than normally aimed for in routine clinical practice and recommended in clinical guidelines. Some described feeling that "sometimes they were asking almost for people to be at too high a risk of hypoglycaemia" (RN17). Others highlighted the lack of evidence of long-term benefits of treating to a tight target: "over a 5 or 6 year period it doesn't seem to improve outcome to any great degree" (Phy3), or commented that "there is some evidence that if you do go too low, it can actually be dangerous" (Phy7). The emphasis placed upon tight blood glucose control rather than other risk factors was also questioned by some staff:

"We probably need to be much tougher on cholesterol, much tougher on blood pressure em, and just do the same as what we're doing for glycaemic control. We've allowed our thinking about this, about how glycaemic control can influence macrovascular events, and they don't really. It doesn't really shape, shape em, it's not going to, it doesn't stop people dying." (Phy5)

Staff also expressed reservations about applying a 'one size fits all' target to all patients commenting that, in clinical practice, treatments would "be individualised to each person" (Phy17). While tight targets were generally seen as appropriate and realistic when patients were relatively young, motivated/adherent and/or at risk of complications, staff described being less keen to pursue tight targets amongst the elderly, especially those who lived alone, and those who did a lot of driving or physical activity:

"A tight target is realistic for anyone who is, its difficult to have a age cut off, but um sort of under the age of 65, who is worried that high glucose levels will damage their blood vessels, and who is up for engaging in better control ... Contrast that with a very old person with lots of other problems in whom you're not going to do them any favours by tightening up their glucose levels. Because you've got to say to yourself, 'well, what are we actually going to achieve here if we make then tight and them falling, and then them being admitted with a fractured hip?'" (RN17)

"Some of them, because of their working lives, you know, they travelled a lot, or they were builders doing really physical work one day, maybe indoors and outdoors and that seemed to vary it. And if they were suddenly having lots of hypos sometimes you were better erring on the side of caution and being higher because at least that way they could still work, still function." (RN19)

As the above quotes suggest, worries "about patient safety" (Phy17) were widely discussed by such staff members. Some staff also raised concerns that increasing patients' risk of hypoglycaemia could potentially undermine trust and the long-term therapeutic relationship: "one hypo and they lose confidence in the system and it's very difficult to do anything with them." (Phy9)

Staff also highlighted the potential dangers of instilling a sense of failure in patients who had not reached the trial target but had achieved better control during 4-T. This informed the decision that some had made to not inform patients of the trial target.

#### Using the TMS

While generally considered a useful guide or starting point for determining patients' insulin doses, all staff, especially those who came to the trial with extensive diabetes clinical experience (see below), described regular deviations from the TMS' recommendations because, "it's for sort of for ideal patients, and not every patient is ideal" (Phy17). In most instances, deviations occurred when, based on their clinical experience, which included that of having previously used the insulins investigated during 4-T, staff considered TMS recommended doses to be too high and as putting a particular patient at risk of hypoglycaemia:

"I think it's difficult really because I've been doing it for x number of years and obviously was very comfortable with those particular insulins. And whether it be a fault of the protocol or the system or just my thinking, I don't know, but it did make you stop and think, 'well that just seems too much.'" (RN13)

In a few cases, deviations were also a direct response to patients' resistance or refusal to have their insulin doses increased after experiencing severe hypoglycaemia. Staff described how this could result in a balancing act "between keeping the patient in the study and trying to fulfil the protocol as best as you could." (RN13)

Some also conveyed concerns about the potentially flawed and 'untrustworthy' data upon which the TMS' recommendations were based. Nursing staff pointed out that the three days of SMBG data fed into the TMS could be a potentially inaccurate representation of a patient's blood glucose control over the previous months:

"But I'm looking back over quite a lot of readings, I would make my decision on that rather than just maybe the week the patient's coming in. Because you have other things, like maybe they've got a stressful week on or, you, you know, you've got the patient in front of you and you've got the diary in front of you, you would sum up and then make your decision." (RN15)

Some also speculated that SMBG data might occasionally have been fabricated by patients; typically when diaries were presented in pristine condition. It was also pointed out that the TMS did not factor in for symptomatic hypoglycaemia above the trial's SMBG 3.1 mmol/l threshold, or for experiences of 'near hypos'. Staff described how they would exercise their own judgement on such occasions, drawing upon their clinical experience:

"Because the thing is, we'd only write hypos in the system, but sometimes patients would be talking about near hypos and obviously there was no way of recording that. So with that in mind, if they said, 'oh, I always feel a touch lower before lunch' blah blah and they told you that quite a bit, there was no way to record that... And then so you'd think 'well I'm going to lower that then, that one, and then put it up a bit more later'. But the TMS didn't know that, so it would tell you different information and then you'd go "oh I'm not happy about that because I think that would drop them down too low." (RN19)

While staff perceived the TMS as a potentially useful tool for less experienced colleagues, such as those based in general practice, some nursing staff said they had disliked using it because they felt it had belittled and undermined their expertise:

"I think for any insulin start trial, you know, you can train a monkey to start insulin, but it takes somebody with more in-depth knowledge to identify where things are going wrong sometimes." (RN2)

Other staff members, however, were more positive about using this technology. They described their previous approach to managing insulin treated patients as having been overly conservative and commented that positive experiences of using the TMS had helped them overcome their resistance to intensifying insulin treatments in both the trial and in their clinical practice:

"We've always been used to rather more tentative doses to start people on. So you know, we would ordinarily have started people on 20 units a day and the slide rule said you start on 40 in the morning and 16 at night or something. Then you're going to step back a little from that and think 'hang on a minute, that seems rather a lot to me'. But once you got used to it, and learnt to trust it, then it seemed to work most of the time." (Phy14)

#### Negotiating the boundary between research and clinical practice

"Working as a DSN and a research nurse, that is a bit of an issue for me... I sometimes found myself in a bit of a dilemma where I think, well off trial, I wouldn't be doing this." (RN2)

As the above quote highlights, cross-cutting the staff interviews is the tension, and difficulties, encountered when the trial protocol required them to deliver patient care which differed from their routine clinical practice. While staff talked about experiencing a conflict between their roles as practitioners and researchers on such occasions, the greatest dilemmas were conveyed by RNs. While this was partly because they were responsible for delivering most direct patient care during 4-T, many RNs also came to the trial with extensive diabetes clinical experience as they had worked and/or were continuing to work part-time in a Diabetes Specialist Nurse capacity alongside 4-T (see table 1). As various staff members commented, the greater the RN's diabetes clinical experience the more likely they were to have deviated from TMS recommended insulin doses during the trial. RN6, for instance, who worked for a research company and did not have a therapeutic speciality, noted that, whilst she had "tended to just go for what was suggested on the computer", her more clinically experienced colleagues:

"were just putting in what THEY thought were the right doses and not going with the algorithm, the protocol." (RN6)

As well as deviating from the TMS' recommendations, clinically experienced RNs also offered patients extra visits and/or input (e.g. dietary advice, training in carbohydrate counting) to those outlined in the protocol, to reflect the care they would provide in their diabetes clinical practice. Some also indicated that they had given patients extra services and enhanced clinical care (such as a quick referral to a chiropody service) to foster treatment adherence and trial retention.

## Discussion

This study sought to understand why there was limited attainment of the primary endpoint (HbA_1c _≤6.5%) in the Treating to Target in Type 2 Diabetes Trial (4-T). Patient interviews did not reveal extensive and intentional acts of treatment non-adherence which would have provided a quick and easy answer to this question. To the contrary, patients generally made efforts to adhere to their insulin regimens during the trial, in some cases by skipping or titrating doses to avoid hypoglycaemia. Some also believed they had done well during the trial despite not reaching the glycaemic target. While previous research has attempted to explain poor adherence to diabetes and other treatments by focusing upon individual patient characteristics, experiences and life circumstances [31], this study highlights the importance of considering the perspectives and experiences of staff. By locating patients' accounts alongside those of 4-T staff, this study has highlighted how staff mediated and buffered patients' experiences of using insulin treatments during the trial, their perceptions of success, and their awareness of the trial target. It has further been shown that the potential impact of staff behaviours on trial outcomes arose in part from the conflict they experienced between their roles and responsibilities as researchers and practitioners and from the requirement to follow a protocol which sought to standardise practice. While they sometimes addressed this conflict by revising their clinical practice in light of trial experiences (e.g. saying they would prescribe larger insulin doses after using the TMS), more frequently, and especially when staff came to the trial with diabetes clinical experience, they adapted the trial protocol to align it with their clinical practices and experiences. This led to instances of staff not following the TMS when recommended doses were perceived as presenting risks to patient trust and safety, or offering patients enhanced care outwith the protocol. While such interventions may have helped to curb or mitigate intentional treatment non-adherence by patients and/or to foster trial retention, they also appear to be a major factor responsible for the limited attainment of the 4-T glycaemic target.

Staff ambivalence about using decision support software and following standardised algorithms has been observed in other healthcare settings, particularly when recommendations contradict professional judgement arising from clinical experience [32,33]. Hence staff attempts to tailor and individualise patient care during 4-T may not simply have been a consequence of working in a trial setting, and may reflect a more general difficulty health professionals encounter when required to follow protocols and clinical guidelines which seek to regulate and standardize their practice [34]. Like this study, research has also found that triallists may struggle to exchange their role of providers of individualised care with that of researchers required to follow standardised trial procedures [25,35], resulting, for instance, in the provision of a 'quasi-clinical service' in the research setting [35,36]. However, while many trials involve new treatments, the insulin therapies compared in 4-T were already widely used in clinical practice. Hence 4-T staff may have had more clinical experience to draw upon than is typical in a trial and may have experienced a greater role conflict as a consequence. Indeed, the data suggest that a large part of staff ambivalence about their participation in 4-T arose from the trial's requirement to treat to a lower target than that routinely used in their clinical practice. Hence the timing of the trial and its location within UK may have impacted on its outcomes. In the UK, management of type 2 diabetes is strongly influenced by the requirements of the UK quality and outcomes framework (QOF). Until recently, the financially incentivised target set by QOF was 7.5%. While the target was reduced to 7% in April 2009 [37], in line with EASD recommendations, it is still higher than that used in 4-T and recommended in other countries [6,7]. Furthermore, it must be noted that, for reasons of patient safety, 4-T staff were allowed a limited degree of leeway in prescribing the dosage as recommended by the TMS. A degree of discretion is often built into protocols of pragmatic trials and complex interventions. However, granting 4-T staff the scope to adjust the TMS' recommendations may have been interpreted by some, particularly the more clinically experienced, as permission to individualise treatments more widely than was originally intended by those who designed the trial.

While the specific context of 4-T delivery needs to be taken into account, this study does raise important issues for the conduct of future trials, especially those, such as 4-T, where staff may be required to follow procedures to assess individual patient safety before implementing mandated therapy changes. Fidelity to protocols is essential for successful trial delivery [38] and this study, alongside others [25,39], highlights the importance of exploring, and addressing, staff perspectives and views. One way to avoid or resolve the role conflict highlighted by 4-T staff and observed elsewhere [35,36] may be to appoint front-line staff who do not have an extensive clinical background or training in the specific area of trial investigation. However, this approach may not be feasible as changes in the research environment have resulted in an increased reliance on nurses working on trials who have primarily clinical rather than research experience. While trial staff might benefit from greater regulation and oversight to foster protocol adherence, such as the recording and feedback of the frequency of their overrides, it has already been shown that these regulatory procedures are unlikely to curb individual decision-making and ensure standardization of practice [33]. Hence multifaceted interventions may be needed to support staff, reduce ambiguity in the courses of action they perceive as open to them [40], and help them 'suspend belief' and provide treatment in accordance with a protocol which may be outside current guidelines of standard clinical care. This may include the provision of more training and education in principles of trial design, aims, and methods, to promote a better understanding of why trial protocols need to be followed and of concepts such as equipoise [25]. Since increasing numbers of clinical staff are becoming involved in clinical trials, this type of education could be made an integral part of all specialist nurse training. Another approach which has already been used to support delivery of trials involving complex interventions is to set aside resources to help promote protocol fidelity. These include extensive training aligned to a manual, use of role play to ensure familiarity with options available in the protocol, recording of interventions, and review of these recordings with a lead facilitator or supervisor to discuss best practice in following the protocol [41,42]. However, this type of approach may be complex and costly to deliver. An alternative is to focus on leadership of research in the clinical setting. One way to do this would be to appoint a cadre of senior research staff who are not directly involved in trial delivery and are available to provide regular support to research nurses. In providing support and supervision, it would be possible to identify issues arising during trial delivery and help resolve them. The building of qualitative work into the early stages of a trial may also be an effective way of identifying staff dilemmas and/or better ways of delivering a trial to promote adherence and retention [43].

A key strength of this study, as already indicated, is that it drew upon the perspectives of staff as well as patients, enabling a clear and comprehensive understanding to be gained of why there was limited attainment of the trial's primary endpoint. Indeed, we would recommend that multiple perspectives (staff and patient) are considered in all future work seeking to understand and/or promote treatment adherence, whether this be in a trial or clinical setting. The use of a multi-site recruitment strategy, which is unusual in qualitative research, increases the potential generalisability of the findings. By using an opt-in procedure (a medical ethics committee requirement), and only interviewing trial completers, the sample may have been biased towards patients with more positive, or extreme, experiences of the trial and injecting insulin. However, this potential limitation was overcome by purposively selecting a patient sample broadly representative of trial participants in terms of glycaemic control and (non-)attainment of the trial's glycaemic target.

## Conclusions

There is a growing trend towards trials which involve the use of licensed treatments, as well as those in which staff are allowed some discretion in exercising or following a protocol (e.g. trials involving complex interventions). As 4-T is a good example of this trend, this study has wide relevance for the rigor with which future trials can be delivered and provides important insights into some of the issues faced by trial staff. The findings highlight that the delivery of trials such as 4-T can be affected by what are currently poorly recognised and debated issues, such as role conflicts experienced by trial staff. These conflicts may be exacerbated when trial protocols are implemented by staff who undertake clinical work alongside, and/or in the same area as, the trial. Our findings suggest that, to promote fidelity to trial protocols, front line staff may benefit from more education, training and support, especially when they are required to provide treatment which is outside current guidelines of standard clinical care. Similarly, involving clinical staff in the development of trial protocols and procedures may help limit the possibility of tensions arising during trial delivery.

## Appendix 1

### Key topics explored in patient interviews

• Treatment history (from diagnosis to referral for insulin).

• Reasons for participating in 4-T; experiences of trial participation (including likes/dislikes of procedures used and guidance received).

• Perceptions and understandings of the trial; perceptions/awareness of trial targets for glycaemic control.

• Reasons for adhering/not adhering to insulin and other treatments during the trial.

• Involvement in treatment changes and dose adjustment decisions.

• Reasons for remaining in the trial; perceived impact of trial participation on diabetes control, general health and quality of life.

### Key topics explored in patient interviews

• Training and background; reasons for involvement in 4-T.

• Perceptions and understandings of the trial and trial target.

• Experiences of initiating and titrating insulin in type 2 patients (in both routine care settings and as part of clinical trials).

• Experiences of delivering 4-T (from initial meetings and patient recruitment to trial close-out); differences between care delivered during 4-T and in routine clinical practice.

• Views about treating to target and using the TMS; reasons for following/not following the TMS's recommendations.

• Reflections on the implications of 4-T findings for clinical practice.

## Competing interests

AF has received funding from the University of Oxford and the NIHR School for Primary Care Research, institutional honoraria from Sanofi Aventis and travel support from Johnson and Johnson; RRH & JLD have declared institutional relationships with Merck, Bayer & Amylin/Lilly alliance, RRH has received payment for board membership activities from Novo Nordisk, Novartis, Merck, Amylin and Lilly and payments for lectures from Bayer, Novo Nordisk, BMS, Novartis, Merck Serono, Ajinomoto and Sanofi Aventis; JL, NH & NJ have no specified relationships with companies that might be relevant to the submitted work.

## Authors' contributions

JL, NH, AJF, JD and RRH designed the study. NJ collected the data. JL and NJ analysed the data. JL drafted the paper. All authors were involved in the critical revisions of the paper and approved the final version. JL is the guarantor.
